# Usability and feasibility of the longitudinal implementation strategy tracking system: a think-aloud study with implementation researchers

**DOI:** 10.3389/frhs.2026.1837215

**Published:** 2026-05-12

**Authors:** James L. Merle, Justin D. Smith

**Affiliations:** Department of Population Health Sciences, Division of Health System Innovation and Research, Spencer Fox Eccles School of Medicine, University of Utah, Salt Lake City, UT, United States

**Keywords:** implementation science, implementation strategies, longitudinal tracking, mixed-methods, research tools, think-aloud, timeline follow-back, usability evaluation

## Abstract

**Introduction:**

Tracking implementation strategies and their adaptations over time remains a persistent methodological challenge in health services research. The Longitudinal Implementation Strategy Tracking System (LISTS) was developed to support structured, longitudinal documentation of strategies, modifications, and discontinuations aligned with established implementation science frameworks using timeline follow-back procedures and an open-access web-based platform. The study objective was to evaluate the usability, feasibility, and methodological fit of the open-access web-based LISTS platform using a structured think-aloud protocol with implementation researchers.

**Methods:**

Nine implementation researchers completed structured think-aloud usability sessions while performing core LISTS tasks, including project setup, strategy entry, bundling, modification logging, discontinuation tracking, and dashboard exploration. Sessions were transcribed and analyzed using hybrid deductive-inductive thematic analysis. Participants also completed the System Usability Scale (SUS) and NASA Task Load Index (NASA-TLX).

**Results:**

The mean SUS score was 76.94, indicating good perceived usability, and mean NASA-TLX workload was low (1.98 on a 1–7 scale). Qualitative findings revealed consistent endorsement of LISTS' structured longitudinal tracking and theoretical alignment with well-known frameworks. Usability barriers included terminology density, multi-level unit hierarchy confusion, and ambiguity in modification-tracking workflows. A cross-cutting tension emerged between theoretical fidelity and accessibility for non-expert users. Despite conceptual friction, participants completed tasks successfully and rated the system positively overall.

**Discussion:**

LISTS demonstrates strong conceptual rigor and functional usability among implementation researchers. However, refinements to terminology scaffolding, hierarchical logic, and modification workflows are needed to enhance accessibility and scalability beyond expert users. Findings highlight broad challenges and considerations for translating implementation science theory and terminology into practical digital infrastructure.

## 1 Introduction

Implementation strategies are the methods used to promote implementation outcomes, such as adoption, fidelity, reach, and sustainment of evidence-based practices (1). Successful implementation depends not only on choosing appropriate strategies but also on clearly specifying, tracking, and reporting how those strategies are deployed and adapted over time (2). Implementation is dynamic; strategies often change in response to contextual factors, stakeholder input, and emerging performance data. Despite this, implementation strategies are frequently underreported, inconsistently specified, and rarely documented longitudinally in a structured manner (2–6). This lack of structured, longitudinal reporting contributes to an explainability gap in implementation science; studies may report improvements in implementation outcomes without clarifying which strategies were used, how strategies were operationalized, or how modifications influenced outcomes. The absence of contemporaneous documentation limits reproducibility and constrains opportunities for cross-site synthesis, and reduces the ability to attribute implementation outcomes to specific strategy components or adaptations.

Several methods have been proposed to track implementation strategies, including activity logs maintained by implementers, coding of implementation team meeting recordings, and multimethod approaches that combine real-time worksheets with periodic qualitative interviews (6–10). Each method offers tradeoffs between precision, burden, and feasibility (11). Activity logs can be low cost but may require *post-hoc* coding. Meeting-based coding reduces burden but may miss details not discussed in meetings. Multimethod approaches can improve completeness but typically require considerable training and resources and remain vulnerable to recall bias when time between events and assessment is lengthy.

The Longitudinal Implementation Strategy Tracking System (LISTS) method (11) was developed to address these limitations by providing a standardized method for assessing, documenting, and tracking implementation strategies over time. LISTS comprises three components: a structured strategy assessment, a data capture platform, and a User's Guide that outlines procedures for routine use. The method draws on existing taxonomies and frameworks, including recommendations for strategy naming and specifying strategy elements (2) and FRAME-IS for documenting adaptations and modifications (12). LISTS also operationalizes a modified timeline follow-back procedure to minimize retrospection bias and to enable frequent, bounded review of strategy use and change (13).

Initial implementation and proof-of-concept of LISTS occurred within a multi-center consortium in which three research centers used the method to support large hybrid effectiveness-implementation trials conducted in healthcare systems (5, 11). In that early evaluation, LISTS was used by research and practice teams for a minimum of 15 months. Results indicated that LISTS is feasible and acceptable as a method for characterizing strategy use and modifications over time. Empirical findings from the proof-of-concept evaluation highlighted both promise and opportunities for improvement. For example, teams reported a mean System Usability Scale score of 67.5 for the REDCap data capture platform used in the proof-of-concept, consistent with a usability grade near the median of established benchmarks. Time investments for initial strategy population and updating varied substantially across centers, with some centers reporting distributed effort on the order of 8 to 21 h for initial population and 1 to 42 h for updates over a 12-month period, depending on study complexity and multisite coordination requirements. These early results underscore that LISTS increases precision and supports common data element approaches, while also indicating the need to streamline procedures and enhance usability for broader adoption.

The REDCap-based LISTS module used in the proof-of-concept included features intended to improve precision and interpretability. These features included auto-population of CFIR constructs (14, 15), pre-populated ERIC strategy categories (1, 16), branching logic to capture planned vs. unplanned modifications, options to designate the level of applicability across units, and a color-coded dashboard to surface active, discontinued, or incomplete entries. Despite these design elements, pilot teams identified practical challenges, including the need for implementation science expertise to resolve nuanced distinctions among taxonomies, the burden of data cleaning and validation, and the requirement for integrated workflows to sustain ongoing documentation.

Given the promising but mixed early evidence, further user-centered refinement of LISTS is warranted. Additionally, due to both use of proprietary features in the original REDCap version of LISTS and the limited dissemination that comes with using REDCap, we undertook the development of a web-based LISTS tool (17). Since it was launched in September, 2024, the LISTS webpage has been accessed by over 1,000 unique users, suggesting growing interest in structured approaches to implementation strategy tracking and supporting the feasibility of broader dissemination. The present study reports a mixed-methods pilot evaluation of an open access web-based LISTS tool and its associated procedures. Using think-aloud cognitive walkthroughs, standardized usability and workload measures, transcript-derived task timing, and protocol feasibility assessment, this study aimed to identify strengths, usability barriers, and high-priority refinements. By integrating quantitative and qualitative evidence, the study seeks to inform iterative development of LISTS as methodological infrastructure for improving the transparency, reproducibility, and synthesis of implementation strategy data across diverse service settings.

## 2 Materials and methods

### 2.1 Study design

This study was conducted as a pilot mixed-methods usability and feasibility evaluation of the Longitudinal Implementation Strategy Tracking System (LISTS) method and online platform. The study had two primary objectives (a) to assess usability, acceptability, and conceptual alignment of the LISTS web-based platform; and (b) to evaluate the feasibility, clarity, burden, and scalability of the structured think-aloud usability protocol intended for use in a future, larger-scale evaluation study. The study employed a concurrent embedded mixed-methods design. Qualitative think-aloud usability testing served as the primary analytic component (18, 19), supplemented by quantitative measures of usability (20), cognitive workload (21), and protocol feasibility. The study was informed by principles of human-centered design and usability science (22).

### 2.2 Participants and recruitment

Participants were recruited using purposive sampling approach, in which individuals were identified through professional networks with experience in implementation research and familiarity with implementation strategy tracking using any method. No formal screening or stratification procedures were used beyond these criteria. This sampling approach was selected to ensure that participants could meaningfully evaluate both the conceptual alignment and practical functionality of the LISTS platform. Twelve individuals meeting these criteria were invited to participate via professional networks and direct email invitation. Nine participants enrolled and completed the usability session (75% participation rate). Participants included early- to mid-career investigators and implementation researchers working in clinical and health system settings. Years of implementation-related experience varied, ensuring representation across differing levels of familiarity with implementation science frameworks and strategy operationalization. Participants were offered compensation at a rate of $150 per hour ($300 total regardless of session length) for their time. Prior use of the LISTS platform was not an explicit exclusion criterion; however, participants were instructed not to interact with the tool prior to the session to preserve the integrity of the usability evaluation.

### 2.3 Pre-Session activities

To ensure baseline familiarity with the LISTS method and platform, participants completed approximately 30 min of structured pre-session activities prior to the usability session. Pre-work included: Review of the LISTS website describing the tool and method (17); optional viewing of a recorded presentation introducing LISTS; review of a standardized vignette describing a fictional Hybrid Type 3 effectiveness-implementation study (23); review of an accompanying data file relevant to the vignette. Participants were instructed not to interact with the LISTS tool prior to the usability session to preserve the integrity of the think-aloud evaluation. The vignette described a multi-site diabetes self-management education (DSME) implementation trial conducted across four clinics within two hospital systems using a staggered rollout design. The scenario included predefined implementation strategies and site-specific adaptations, including strategy additions, modifications, and discontinuations. All participants confirmed completion of pre-session materials, including review of the LISTS website and viewing of the recorded presentation. The recorded presentation was approximately 16 min in duration.

### 2.4 LISTS tool overview

LISTS is an open-access web-based application (https://hivimpsci.northwestern.edu/longitudinal-implementation-strategy-tracking-system/) designed to support structured, longitudinal tracking of implementation strategies (17). Core features include:

- Project setup with specification of actors and multi-level organizational units
- Entry of discrete implementation strategies
- Strategy bundling for multi-component or meta-strategies
- Documentation of start and stop dates
- Logging of strategy modifications and discontinuations
- Customizable dashboard visualization and filtering using a Gantt-style timeline
- Export of data in tabular and image formats

The platform incorporates terminology and constructs from established implementation science taxonomies, models, and frameworks (2, 12, 16, 24–29). Representative screenshots of key LISTS interface components corresponding to the core functionalities are provided in Supplementary File 1 to support understanding of the tool and usability tasks.

### 2.5 Usability session procedures

Each participant completed a single, structured usability session lasting approximately 60–90 min via videoconference. Sessions included audio recording and screen sharing to capture real-time interaction with the tool.

#### 2.5.1 Think-Aloud protocol

During navigation to each core function of the tool, participants were instructed to verbalize: (a) what they were doing; (b) why they were making specific selections; (c) points of confusion or hesitation; (d) reactions to terminology and interface structure; and (e) recommendations for improvement. The facilitator prompted continued, stream-of-consciousness verbalization when necessary but minimized direct assistance during task completion. Clarification was provided only when participants were unable to proceed.

#### 2.5.2 Standardized task sequence

Participants completed a standardized sequence of tasks using the provided vignette:

1. Project setup, including entry of project information and multi-level organizational units.
2. Entry of a discrete implementation strategy mapped to ERIC categories and CFIR determinants
3. Creation of a bundled strategy.
4. Exploration of dashboard visualization, filtering, and export functions.
5. Logging of a mid-study strategy modification affecting a specific unit.
6. Documentation of strategy discontinuation.

These tasks correspond to core interface components illustrated in Supplementary File 1. The standardized vignette and data file ensured consistency across sessions, enabling cross-case comparison.

### 2.6 Quantitative measures

Following task completion, participants completed structured questionnaires assessing system usability, cognitive workload, and protocol feasibility.

#### 2.6.1 System usability scale (SUS)

The System Usability Scale (SUS) is a 10-item instrument assessing perceived usability on a 5-point Likert scale (20). Items assess complexity, ease of use, integration, and user confidence. SUS scores range from 0 to 100, with a score of 68 commonly considered average usability. Scores between 70 and 79 are generally interpreted as “good,” scores ≥80 as “very good” to “excellent,” and scores below 60 as indicative of marginal usability (30).

#### 2.6.2 NASA task load Index (NASA-TLX)

Perceived cognitive workload was assessed using the NASA Task Load Index (NASA-TLX), a widely used instrument for evaluating subjective workload during task performance (21). The NASA-TLX measures workload across multiple domains using Likert-type scales, with higher scores indicating greater perceived workload. In this study, five workload domains were assessed. *Mental demand* reflects the degree of cognitive activity required, including thinking, decision-making, remembering, and problem-solving. *Temporal demand* captures the extent to which participants experienced time pressure while completing tasks. *Performance* reflects participants' self-evaluation of how successfully they completed the tasks. *Effort* represents the amount of work participants perceived they had to expend to accomplish the tasks. *Frustration* assesses the degree of annoyance, stress, or irritation experienced during task completion. NASA-TLX scores range from 1 to 7 for each workload domain, with lower scores indicating lower perceived workload. In usability evaluations of digital systems, mean scores near 1–2 typically reflect low cognitive burden, whereas scores above 4 suggest moderate to high workload (31).

#### 2.6.3 Protocol feasibility assessment

In addition to evaluating usability of the LISTS platform, participants completed a structured protocol feasibility assessment at the conclusion of the usability session. The purpose of this assessment was to evaluate the acceptability, clarity, and scalability of the think-aloud testing protocol itself, as this study was designed as a pilot feasibility evaluation intended to inform a larger future study. The protocol feasibility instrument included Likert-scale items assessing participants' perceptions of the clarity of instructions, appropriateness of the vignette, alignment between vignette tasks and tool functionality, cognitive burden of the testing procedures, and overall feasibility of implementing the protocol in a larger study. Participants also rated whether the overall session length was acceptable and whether the protocol could reasonably be replicated or scaled. Items were rated on a 0–3 scale, with higher scores indicating greater agreement with statements reflecting clarity, acceptability, and feasibility. Descriptive statistics were calculated for each item and for the overall feasibility score. Given the pilot and formative nature of the study and small sample size (*N* = 9), results were interpreted descriptively and used to inform refinement of the usability testing procedures. Open-ended responses solicited recommendations for protocol refinement and anticipated barriers to broader implementation.

### 2.7 Data management

Sessions were transcribed verbatim using automated captioning software and reviewed for accuracy. Identifying information was removed prior to analysis. De-identified transcripts were stored on secure institutional servers.

### 2.8 Qualitative analysis

We employed a hybrid deductive–inductive thematic analysis approach. Deductive codes were informed by usability science domains (e.g., navigation, learnability, error prevention) (19, 22), implementation outcomes (e.g., acceptability, feasibility, appropriateness) (29), and anticipated functional domains (e.g., unit hierarchy, strategy modification tracking, visualization). These domains were used to guide initial coding categories, while allowing for inductive refinement based on participant responses. Inductive coding captured emergent themes related to conceptual alignment, terminology burden, workflow integration, and protocol scalability. All transcripts were reviewed in full prior to coding. Segments reflecting usability strengths, usability barriers, cognitive load indicators, workflow fit, and protocol feasibility were coded. Codes were iteratively refined through constant comparison across transcripts. A cross-case matrix was developed to identify recurring themes and areas of divergence. The full analytic codebook, including operational definitions and inclusion/exclusion criteria, is provided as Supplementary Table S1.

#### 2.8.1 Task duration estimation

To characterize task-level workload during usability testing, task completion times were estimated using transcript timestamps. Start times were defined as the moment the facilitator explicitly prompted a participant to begin a task (e.g., “please fill in…,” “add a new strategy,” “track the modification”). End times were defined as the moment the facilitator closed the task (e.g., “good job,” “let's move on,” or instructions to proceed to the next task). Five structured tasks were included in the analysis: project setup, adding a strategy, bundling strategies, tracking a modification, and tracking a discontinuation. Dashboard exploration was excluded due to its open-ended nature. Durations were calculated as the difference between task start and end timestamps. Mean task durations were calculated across participants with valid timing data. Given the pilot nature of the study and small sample size (*N* = 9), results are interpreted descriptively.

#### 2.8.2 Thematic sufficiency assessment

To assess thematic sufficiency, cross-case comparison was conducted throughout analysis. Following initial coding of early transcripts, emergent themes were iteratively compared against subsequent cases to identify novel categories or substantive deviations. Attention was given to whether later interviews introduced new primary usability domains or instead elaborated previously identified themes. After analysis of the final two transcripts, no substantively new primary themes emerged, and observed variation reflected elaboration rather than category expansion. Given the focused usability objectives and relatively homogeneous participant pool, thematic sufficiency was determined based on recurrence and stability of core categories rather than formal saturation metrics.

### 2.9 Quantitative analysis

SUS and NASA-TLX scores were summarized using descriptive statistics (means, standard deviations, and ranges). Protocol feasibility item responses were summarized descriptively. Given the exploratory and formative nature of the study and small sample size (*N* = 9), inferential statistical testing was not performed. Quantitative findings were integrated with qualitative themes to contextualize perceptions of usability and feasibility.

### 2.10 Mixed-Methods integration

Mixed-methods integration was conducted using a concurrent triangulation approach (32). Qualitative and quantitative data were analyzed separately and then integrated during interpretation to examine convergence, complementarity, and divergence across data sources. Quantitative measures (SUS and NASA-TLX) were used to characterize overall usability and perceived workload, while qualitative think-aloud data provided insight into user experiences, sources of friction, and recommendations for improvement. Integration occurred through iterative comparison of quantitative results with qualitative themes. Specifically, qualitative findings were used to contextualize and explain quantitative usability and workload scores, while quantitative findings were used to assess the extent to which identified usability challenges were associated with perceived burden or task difficulty. Transcript-derived task duration estimates were also incorporated to triangulate areas of increased cognitive engagement. A joint display approach (33) was used conceptually to align qualitative themes with quantitative indicators (e.g., SUS scores, NASA-TLX ratings, and task durations), enabling identification of patterns of convergence (e.g., low workload despite reported conceptual friction) and complementarity (e.g., qualitative explanations for longer task durations). Integration focused on identifying whether usability barriers reflected structural interface limitations or interpretive challenges related to implementation science constructs.

### 2.11 Ethical considerations

This study was reviewed by the University of Utah Institutional Review Board and determined to qualify for exemption from formal IRB review as minimal-risk research. All participants provided informed consent prior to participation. Participants were offered compensation at $150 per hour for their time. All data were de-identified prior to analysis.

## 3 Results

### 3.1 Participant characteristics

Nine participants completed the usability sessions. Participants were implementation researchers working in community, clinical and health system settings and represented a range of academic ranks and years of implementation-related experience (Table 1). All participants conducted research in clinical or health system settings, with additional representation in community-based settings (*n* = 4*,* 44.4%), Veterans Affairs (VA) systems (*n* = 1*,* 11.1%), faith-based organizations (*n* = 1, 11.1%), and rural contexts (*n* = 1, 11.1%). Participants had a mean of 8.1 years of implementation research experience (median = 7, range = 4–20).

**Table 1:** Participant demographics.

Characteristic	Value
Total Participants	9
Academic Rank	
Assistant Professor	5 (55.6%)
Associate Professor	2 (22.2%)
Full Professor	2 (22.2%)
Years in Current Role	*Mean* = 4.00 years (*SD* = 4.18)
	*Median* = *2* (*range* = 1–12)
Years of Implementation	*Mean* = 8.11 years (*SD* = 5.47)
Research Experience	*Median* = *7* (*range* = 4–20)
Primary Research Contexts*	
Clinical/Health System Settings	9 (100%)
Community Settings	4 (44.4%)
Veterans Affairs (VA) Settings	1 (11.1%)
Faith-Based Organizations (FBO)	1 (11.1%)
Rural Settings	1 (11.1%)

*Participants could report multiple research contexts; total percentages therefore exceed 100%.

### 3.2 Quantitative findings

Quantitative usability findings are summarized in Table 2. The mean System Usability Scale (SUS) score was 76.94 (SD = 13.33), indicating good overall usability. Scores exceeded the conventional acceptability benchmark of 68 (30, 34), suggesting that participants perceived the LISTS platform as usable despite identified areas of refinement. The mean overall NASA Task Load Index (NASA-TLX) score was 1.98 (SD = 0.67) on a 1–7 scale, reflecting low perceived cognitive workload. This indicates that while participants described conceptual density in qualitative interviews, the tool did not impose substantial mental or temporal strain during task completion. The average protocol feasibility rating across items was 2.74 (SD = 0.28) on a 0–3 scale, indicating high perceived feasibility of the think-aloud procedure and vignette-based task structure. Although minor variability was observed in items related to session length and scalability, overall ratings supported the acceptability of the pilot protocol.

**Table 2:** Quantitative results.

Measure	Scale	Mean	SD	Min	Max	Interpretation
System Usability Scale (SUS)	0–100	76.94	13.33	57.50	100.00	Good usability
NASA-TLX (Overall Workload)	1–7	1.98	0.67	1.20	3.40	Low workload
Protocol Feasibility (Average Across Items)	0–3	2.74	0.28	2.20	3.00	High feasibility

#### 3.2.1 Task duration findings

Transcript-derived timing estimates indicated variation across tasks. Mean completion times were as follows: project setup (8.45 min), tracking a modification (7.46 min), adding a strategy (6.97 min), bundling strategies (2.19 min), and tracking a discontinuation (1.87 min). Tasks involving conceptual specification, particularly adding a strategy and tracking a modification, required the greatest engagement time. In contrast, bundling and discontinuation tracking required comparatively less time once participants were oriented to the system.

### 3.3 Qualitative findings: tool usability

#### 3.3.1 Strength: structured longitudinal strategy tracking

The most consistently endorsed strength was LISTS' ability to systematically track implementation strategies and adaptations over time. Participants described this as filling an important methodological gap in implementation science. Several participants emphasized that longitudinal documentation of modifications and discontinuations is critical for understanding what “actually works” in implementation research. The structured logs and timeline view were described as especially valuable for retrospective analysis and transparency. The ability to document modifications and discontinuations in a structured, auditable format was viewed as addressing a persistent gap in implementation research methodology.

#### 3.3.2 Strength: conceptual alignment with implementation frameworks

Participants noted that LISTS demonstrates strong alignment with established implementation science theories, models, frameworks, and taxonomies. This alignment was viewed as enhancing methodological rigor and ensuring conceptual consistency within implementation research.

#### 3.3.3 Strength: dashboard visualization and export functionality

The dashboard interface and timeline visualization were generally perceived as intuitive and useful for monitoring implementation activity over time. Export functionality was viewed as particularly valuable for communication with research teams and stakeholders.

#### 3.3.4 Limitation: terminology density and accessibility

A prominent limitation across participants was the density of implementation science terminology embedded within the interface. This was evident during both concurrent task performance and post-session reflection. Although participants recognized the theoretical validity of distinctions such as sustainment vs. sustainability, many expressed concern that such terminology may limit usability for project managers or implementation-adjacent users. Importantly, this concern was not framed as disagreement with the constructs themselves, but rather as a barrier to accessibility.

#### 3.3.5 Limitation: multi-level unit hierarchy confusion

Participants frequently reported confusion during project setup when specifying multi-level organizational units. The distinction between hierarchical levels was not consistently intuitive, particularly in multi-site scenarios involving nested health systems and clinics.

#### 3.3.6 Limitation: ambiguity between editing and logging modifications

Several participants had trouble distinguishing between editing original strategy information and logging a modification. This ambiguity raised concerns regarding the preservation of an audit trail and the potential for unintentional overwriting of original strategy definitions.

#### 3.3.7 Cross-Cutting theme: fidelity versus accessibility

Across interviews, a central analytic tension emerged between theoretical fidelity and practical accessibility. While participants consistently valued LISTS' alignment with established frameworks, this rigor was perceived as contributing to terminology density and interface complexity. Participants indicated that broader adoption may require simplified modes or additional scaffolding for non-expert users.

#### 3.3.8 Thematic sufficiency and cross-case consistency

Given the focused usability objectives and relatively homogeneous participant pool (implementation researchers with experience in strategy tracking), thematic sufficiency was assessed through cross-case comparison rather than formal saturation metrics. Core themes, including terminology density and perceived value of structured longitudinal tracking, were present across all nine transcripts. Additional primary usability barriers, including multi-level unit hierarchy confusion and ambiguity between editing and logging modifications, were observed in most cases. No substantively new primary themes emerged in the final two interviews; rather, later sessions reinforced and elaborated previously identified categories. These patterns suggest that thematic sufficiency was achieved for the study's targeted usability and feasibility objectives, consistent with established usability research practices emphasizing task-focused analytic depth (18, 19). A cross-case thematic matrix is provided in Supplementary Table S2.

### 3.4 Recommendations for improvement

Across both in-session navigation and post-SUS reflection, participants identified terminology density as a primary usability barrier. During strategy entry tasks, participants hesitated when selecting outcomes and determinants, particularly when distinguishing among closely related constructs. In post-session reflections, participants emphasized that the tool may be difficult for users without formal implementation science training. Participants recommended simplifying terminology, collapsing overlapping constructs where appropriate, or developing a “LISTS Lite” version that provides plain-language descriptors while preserving theoretical rigor for expert users. Participants recommended clearer labeling, more obvious visual indentation, or scaffolding to reduce confusion during multi-site setup. Importantly, these recommendations emerged both during active use of dropdown menus and in global reflections about scalability, suggesting that terminology density represents a structural accessibility issue rather than a momentary navigation problem.

### 3.5 Protocol feasibility

Participants generally found the think-aloud protocol clear and effective for eliciting usability feedback. The vignette and pre-work materials were perceived as appropriate and comprehensive. However, several participants noted that session length (60–90 min) may present scalability challenges for larger studies. Participants suggested modularizing tasks or shortening sessions for broader implementation. Overall, the protocol was considered feasible for pilot evaluation but likely to require refinement prior to use in a large-scale study.

### 3.6 Mixed-methods integration

Integration of quantitative and qualitative findings revealed a nuanced usability profile of the LISTS platform. Quantitatively, LISTS demonstrated good overall usability (mean SUS = 76.94) and low perceived cognitive workload (mean NASA-TLX = 1.98). These findings indicate that participants were able to complete tasks successfully and without substantial mental strain. However, qualitative findings revealed consistent conceptual friction points, particularly related to terminology density, hierarchical unit specification, and modification-tracking workflows.

Importantly, the low NASA-TLX scores suggest that these usability challenges did not reflect overwhelming cognitive burden or task failure. Rather, they reflect interpretive and conceptual friction. Participants were able to navigate the interface, but reported needing to pause, reinterpret terminology, or reconcile conceptual distinctions while doing so.

Transcript-derived task timing estimates reinforce this interpretation. Tasks requiring conceptual specification, particularly adding a strategy (mean = 6.97 min) and tracking a modification (mean = 7.46 min), required two of the three longest engagement times. During modification tracking, several participants expressed uncertainty about whether they were required to update multiple fields, even when only one element of the strategy had changed. Some participants attributed this confusion to the visual design of the modification interface, specifically the use of orange-colored text, which appeared to signal that additional fields required completion. Although participants ultimately completed the task correctly, this momentary hesitation illustrates how visual affordances may amplify perceived complexity without increasing actual workload. In contrast, bundling (mean = 2.19 min) and discontinuation tracking (mean = 1.87 min) were completed more quickly once participants were oriented to the system. Project setup required a mean of 8.45 min, reflecting deliberation around hierarchical unit specification.

Thus, quantitative workload ratings, qualitative feedback, and task-level engagement converge in suggesting that LISTS is structurally usable but conceptually dense. Friction emerged primarily when participants interpreted framework terminology or responded to interface cues that implied additional cognitive steps, rather than from navigation difficulty itself.

The moderate-to-high SUS scores further contextualize qualitative concerns. Despite identifying several barriers, participants still rated the tool positively overall. This pattern suggests that participants perceived the system as fundamentally sound and valuable, even while recognizing areas for refinement. In other words, usability challenges did not undermine overall system credibility.

A key mixed-methods insight emerging from integration is that the primary usability limitations are not rooted in interface mechanics, but in the translation of implementation science theory into operationalized digital workflows. The central tension between fidelity and accessibility helps explain why usability ratings were strong while conceptual density remained a concern. LISTS successfully operationalizes implementation science frameworks, but that success introduces complexity that may impede broader adoption. Protocol feasibility findings reinforce this interpretation. Participants rated the think-aloud protocol as highly acceptable (mean feasibility = 2.74/3), and NASA-TLX scores indicate that the evaluation process itself did not impose high burden. However, qualitative reflections suggested that session length may limit scalability. This convergence indicates that the protocol is methodologically sound but may benefit from efficiency refinements prior to larger-scale deployment.

Overall, the integrated findings suggest that LISTS is a conceptually rigorous and functionally usable tool that would benefit from targeted refinements aimed at improving accessibility, clarifying hierarchical logic, and strengthening modification-tracking workflows. The combination of good usability scores, low workload ratings, and longer engagement times for conceptually demanding tasks supports progression to iterative refinement rather than fundamental redesign.

## 4 Discussion

### 4.1 Principal findings

This pilot mixed-methods evaluation examined the usability and feasibility of the Longitudinal Implementation Strategy Tracking System (LISTS) and its associated think-aloud protocol. Overall, LISTS demonstrated good perceived usability (mean SUS = 76.94) and low cognitive workload (mean NASA-TLX = 1.98), while qualitative findings identified targeted areas for refinement. Participants consistently endorsed LISTS' structured longitudinal tracking capabilities and alignment with established implementation science frameworks. At the same time, recurring usability barriers, including terminology density, hierarchical unit specification, and ambiguity in modification-tracking workflows, were identified as priority areas for improvement.

Importantly, integration of quantitative and qualitative findings revealed that usability challenges were primarily conceptual rather than mechanical. Despite reporting terminology density and occasional interface friction, participants were able to complete tasks successfully and without substantial mental burden. This divergence suggests that the LISTS online platform, is structurally usable but conceptually dense. The conceptual density is not limited to the platform as the LISTS method itself is likely to be viewed as conceptually dense.

### 4.2 Fidelity versus accessibility

A central cross-cutting theme emerging from this study was the tension between theoretical fidelity and accessibility. Participants strongly valued LISTS' close alignment with implementation science frameworks, including ERIC (1), CFIR (15), and RE-AIM (28) and Proctor implementation outcomes (29, 35). This alignment enhances methodological rigor and supports standardized reporting. However, participants also noted that this conceptual precision contributes to terminology density and may limit accessibility for implementation-adjacent users, such as project managers, who may be tasked with conducting LISTS data entry and management. Notably, this concern was not framed as disagreement with the constructs themselves. Rather, it reflects a broader challenge in operationalizing implementation science theory within user-friendly digital systems.

The quantitative findings reinforce this interpretation. The relatively high SUS score indicates that participants perceived the tool as usable overall, while low NASA-TLX scores suggest that terminology density did not translate into excessive cognitive strain. Together, these findings suggest that targeted refinements, such as simplified terminology options or scaffolded guidance, may improve accessibility without sacrificing theoretical integrity.

### 4.3 Method-Level implications for implementation strategy tracking

Participants' feedback extended beyond interface usability to the broader LISTS method. Several participants emphasized that challenges in strategy tracking originate upstream of tool use. Specifically, difficulty operationalizing discrete implementation strategies across sites was identified as a persistent field-level issue. LISTS makes these ambiguities visible, rather than creating them.

Participants also highlighted the behavioral complexity of near-real-time adaptation tracking. Clinicians and frontline staff may have limited time to document strategy modifications, and adaptation decisions are often communicated informally. These insights suggest that successful implementation of LISTS requires deliberate integration into existing workflows, such as structured implementation team meetings or recurring check-ins. At the same time, the LISTS method incorporates a timeline follow-back approach designed to mitigate some of these challenges by supporting structured, interval-based reporting of implementation strategies and adaptations (11). Rather than requiring continuous real-time documentation, this approach allows users to record strategy use and changes within defined time windows, using temporal anchors to support recall while minimizing retrospection bias (13). This design may reduce burden on clinicians and implementation teams who may not be able to consistently document changes as they occur in dynamic practice settings, while still maintaining a structured and systematic record of strategy evolution. Although the present study did not directly evaluate the application of timeline follow-back procedures in real-world implementation contexts, this feature represents a potential methodological strength of LISTS for balancing feasibility and precision in longitudinal strategy tracking. Future research should examine how this approach performs in practice, including its impact on data completeness, accuracy, and workflow integration.

Additionally, participants suggested that effective use of LISTS may require oversight by individuals with formal training in implementation science. This raises important considerations for scalability and user targeting in future development phases. Together, these findings indicate that refinement of LISTS must address both interface-level usability and method-level integration within real-world implementation contexts.

#### 4.3.1 Task-Level engagement and conceptual workload

Task duration findings provide additional insight into how participants interacted with LISTS. Although overall cognitive workload ratings were low (mean NASA-TLX = 1.98), tasks requiring conceptual interpretation, particularly strategy entry and modification tracking, required the longest engagement time. This pattern suggests that participants were not cognitively overwhelmed but rather engaged in interpretive processing related to framework alignment and strategy specification. In contrast, bundling and discontinuation tasks were completed more quickly, indicating that once strategies were defined, structural actions within the interface were relatively straightforward. Importantly, the alignment between longer task durations and qualitatively reported conceptual friction reinforces the interpretation that usability challenges are rooted primarily in theoretical density rather than interface mechanics. These findings further support the need for terminology scaffolding and clearer modification workflows.

It is also likely that task completion time would decrease with repeated use as users become more familiar with the interface and underlying workflow. As with many complex data capture systems, initial interactions may require greater cognitive effort, whereas subsequent use may reflect increased efficiency through learning and routinization. In addition, participants completed tasks using a standardized vignette, which introduced a layer of unfamiliarity with the project context and strategy details that may have contributed to slower task performance independent of the tool itself. However, the consistent alignment between longer task durations and conceptually demanding tasks suggests that these patterns are not solely attributable to learning effects or vignette-related unfamiliarity, but also reflect the inherent complexity of specifying and tracking implementation strategies within structured frameworks.

### 4.4 Protocol feasibility and scalability

The structured think-aloud protocol was rated as highly feasible (mean feasibility score = 2.74/3). Participants found the vignette realistic and the task sequence comprehensive. NASA-TLX scores indicate that the protocol did not impose substantial cognitive burden. However, participants identified session length (60–90 min) as a potential scalability limitation. Modularizing tasks or tailoring sessions to specific user roles may improve feasibility in larger studies. These findings support progression to broader testing while refining protocol efficiency.

### 4.5 Implications for health services research

Longitudinal tracking of implementation strategies remains a persistent methodological challenge in health services research. Although implementation frameworks provide conceptual structure, operationalizing strategy tracking in practice is often inconsistent and retrospective. LISTS represents an effort to translate implementation science theory into structured digital infrastructure. This study demonstrates that such translation is feasible and well-received by expert users, but it requires careful attention to accessibility and workflow integration. The findings underscore that development of implementation tools should incorporate usability science and mixed-methods evaluation early in the design process. Iterative refinement grounded in user feedback may help bridge the gap between theoretical rigor and real-world adoption.

### 4.6 Limitations

This study has several limitations. First, the sample consisted primarily of implementation researchers with prior familiarity with implementation frameworks. Usability perceptions may differ among implementation-adjacent users or frontline staff. Second, sessions were conducted in a simulated vignette context rather than within an active implementation trial. Third, task duration estimates were derived from transcript timestamps and therefore reflect combined navigation and discussion time rather than pure system interaction time. As a pilot feasibility study, the sample size was small (*N* = 9), and quantitative findings were interpreted descriptively. Finally, as participants were compensated and recruited through professional networks, social desirability bias or simply sampling bias of positively predisposed users cannot be ruled out.

### 4.7 Future directions

Future work should include iterative refinement of high-priority usability areas, including terminology scaffolding, unit hierarchy clarity, and modification-tracking workflows. Subsequent evaluation phases should expand testing to include implementation-adjacent users and real-world multi-site implementation studies both within and outside of health services research settings. Additionally, integration strategies, such as embedding LISTS into recurring implementation meetings or developing streamlined capture workflows, should be explored to enhance sustainability of adaptation tracking.

## 5 Conclusion

In this pilot mixed-methods evaluation, the LISTS online tool demonstrated good usability and low cognitive workload among implementation researchers. Participants strongly endorsed the platform's structured longitudinal tracking and framework alignment. Usability refinements focused on improving accessibility and clarifying hierarchical and modification workflows are warranted. These findings support continued iterative development and broader testing of LISTS as infrastructure for advancing implementation strategy tracking in health services research.

## Data Availability

The raw data supporting the conclusions of this article will be made available by the authors, without undue reservation.

## Supplementary material

Supplementary File 1 Representative screenshots of the LISTS tool used for the six usability tasks.

Supplemnetary Table S1 Codebook for Hybrid Deductive–inductive thematic analysis.

Supplementary Table S2 Cross-case matrix for primary qualitative themes.
