# Supplementary material for: Usability and feasibility of the longitudinal implementation strategy tracking system: a think-aloud study with implementation researchers
Source: Front Health Serv. 2026 May 12;6:1837215. doi: 10.3389/frhs.2026.1837215 (PMC13201223; doi:10.3389/frhs.2026.1837215)
Supplement: Supplementary File 1 — Representative screenshots of the LISTS tool used for the six usability tasks. [file Supplementaryfile1.docx]

**Supplemental Figures Table of Contents**

**Representative screenshots of the LISTS tool used for the six usability tasks**

Supplemental Figure 1: Project Set-Up

Supplemental Figure 2: Adding Strategies

Supplemental Figure 3: Bundling Strategies

Supplemental Figure 4: Dashboard

Supplemental Figure 5: Strategy Information and Tracking

Supplemental Figure 6: Tracking Strategy Modifications


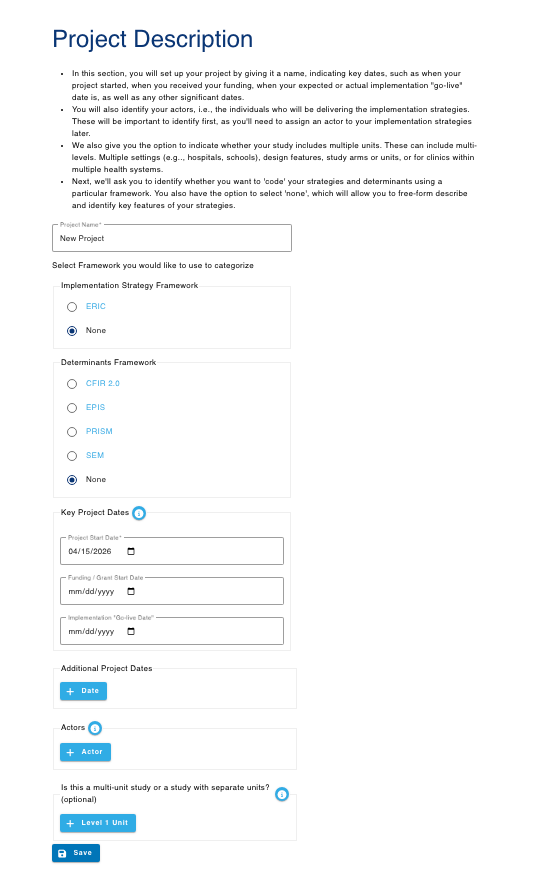
Supplemental Figure 1. Project Set Up Page

*Note*. Representative screenshot of the Project Set-Up page, where users enter key project-level information, including project name, selected frameworks, key dates for timeline display, actors responsible for delivering strategies, and hierarchical organization of study units across up to four nested levels.


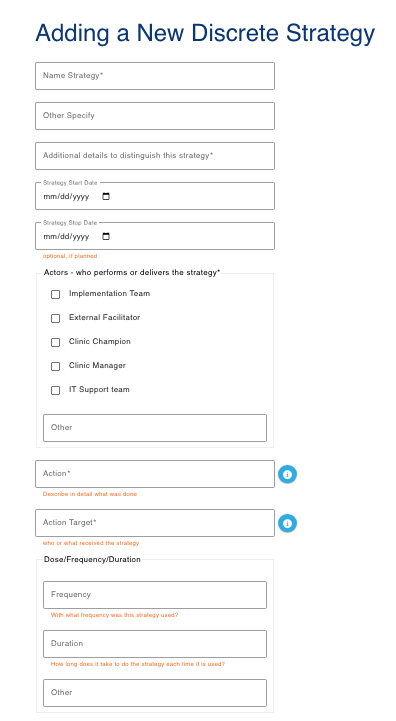
Supplemental Figure 2. Adding a Strategy


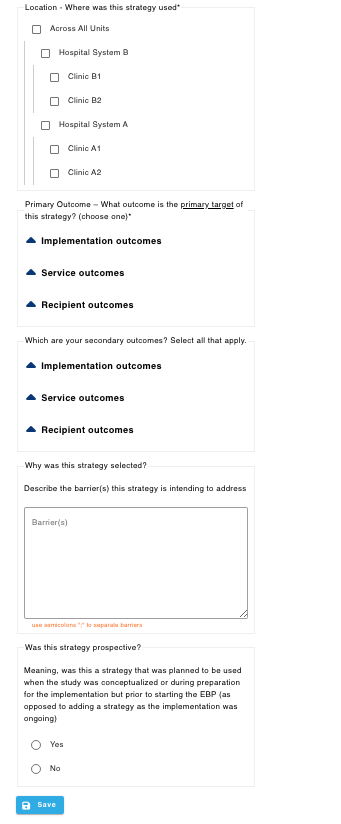
*Note.* Representative screenshot of the Add Strategy page, where users define individual implementation strategies by specifying the strategy name, description, start and stop dates, actors, actions, targets, dose, outcomes, and justification. Additional fields allow assignment of ERIC strategy categories and relevant implementation determinants or contextual factors (not shown).

Supplemental Figure 3. Bundling Strategies


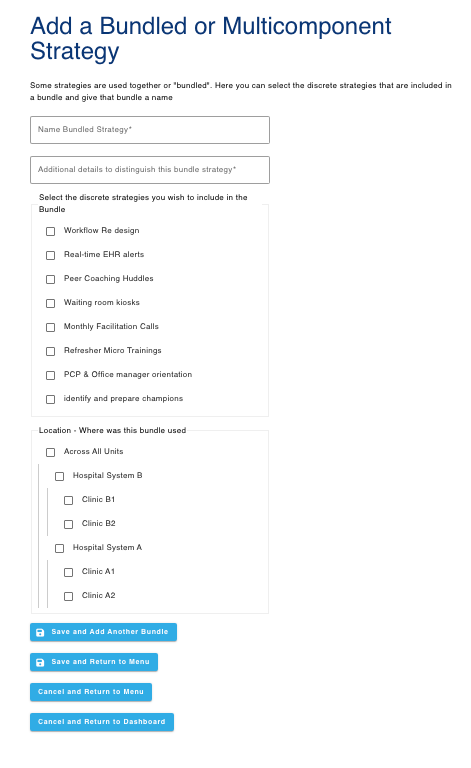


*Note*: Representative screenshot of the Bundling Strategies page, where users group related or multicomponent strategies to support organization and visualization within the dashboard. (see Supplemental Figure 4).


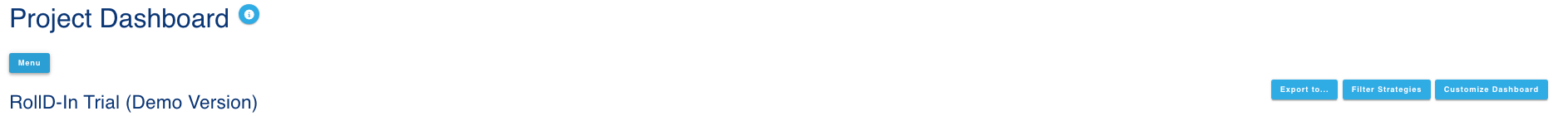

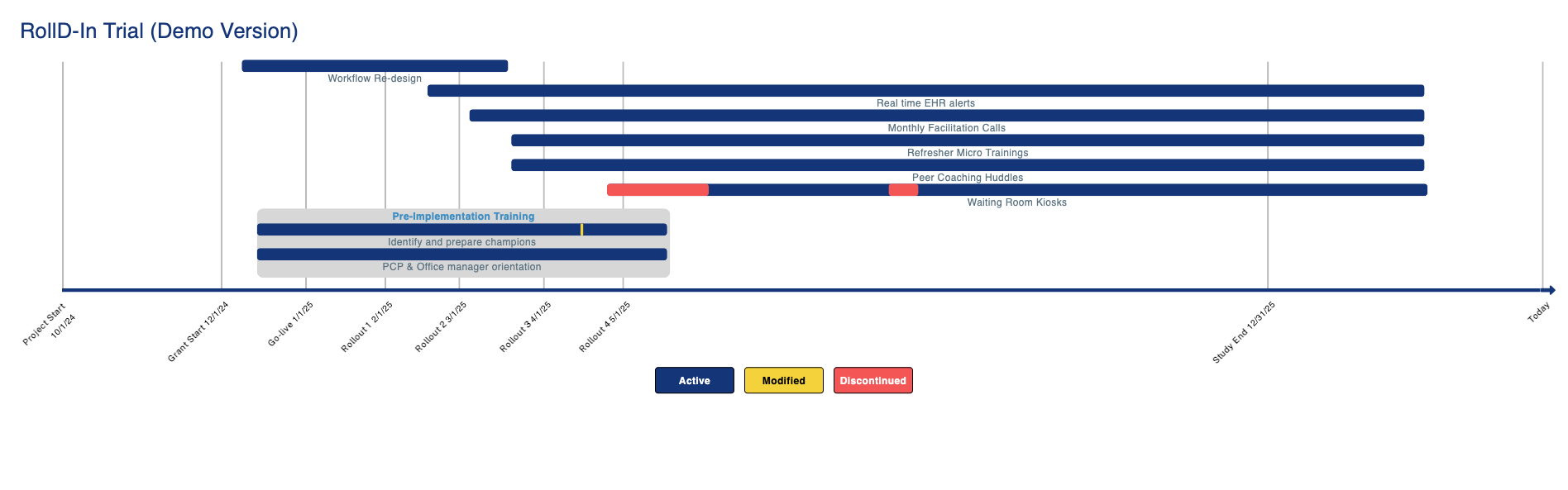
Supplemental Figure 4. Example Project Dashboard

*Note*. Representative screenshot of the interactive project dashboard. Each entered strategy is displayed as a horizontal bar on a timeline. This page is interactive; users can click on strategies and be shown each strategy’s information (see supplemental figure 5). Bundled strategies are identified by the light grey box encompassing individual or discrete strategies. Blue areas on the bars indicate periods where the strategy was active; red areas indicate when the strategy was discontinued or turned off; and yellow slashes indicate tracked modifications that align with the date on the timeline. Using the “Filter Strategies” button, strategies can be filtered to show only strategies associated with particular units or sites, outcomes, whether they were prospective, etc. The “Export to…” button allows for exporting an image file of the dashboard as well as a .csv file of the entered data. Finally, the “Customize Dashboard” button allows users to reorganize the strategies on the dashboard or align them automatically by start date.

Supplemental Figure 5. Strategy Information Page


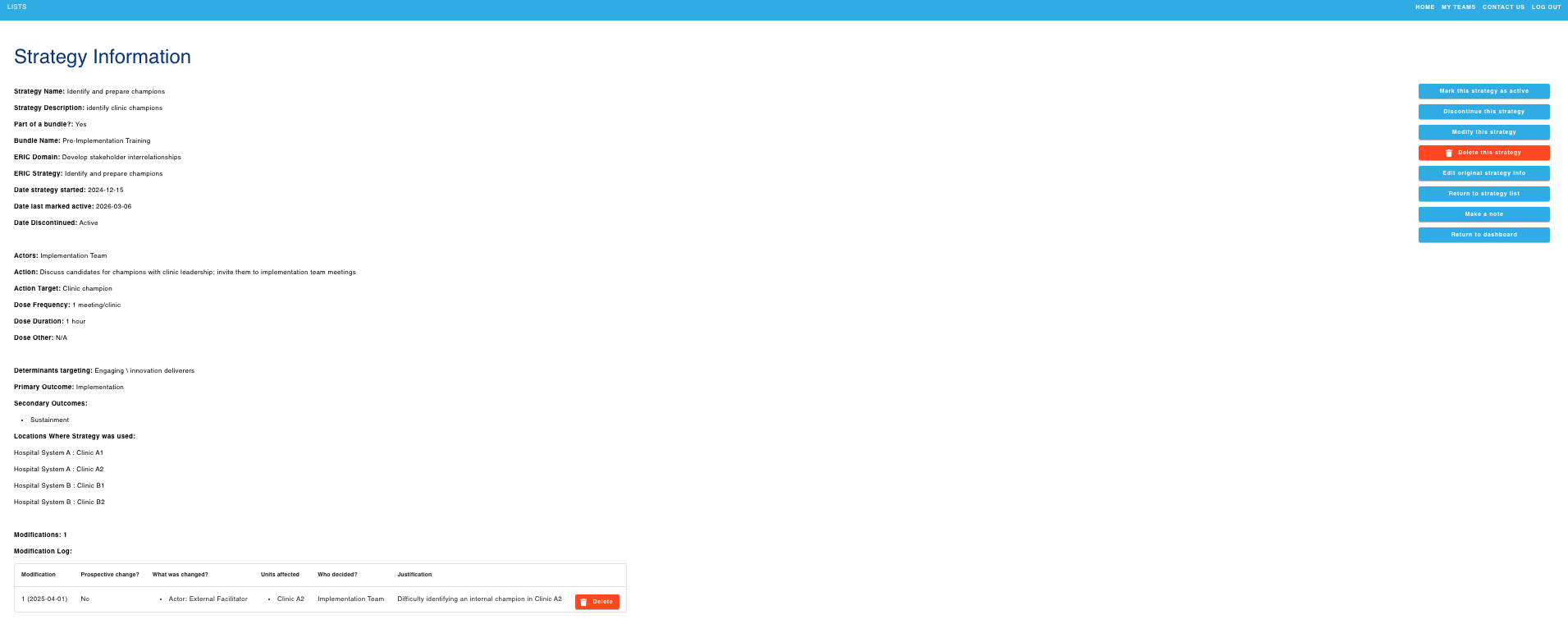

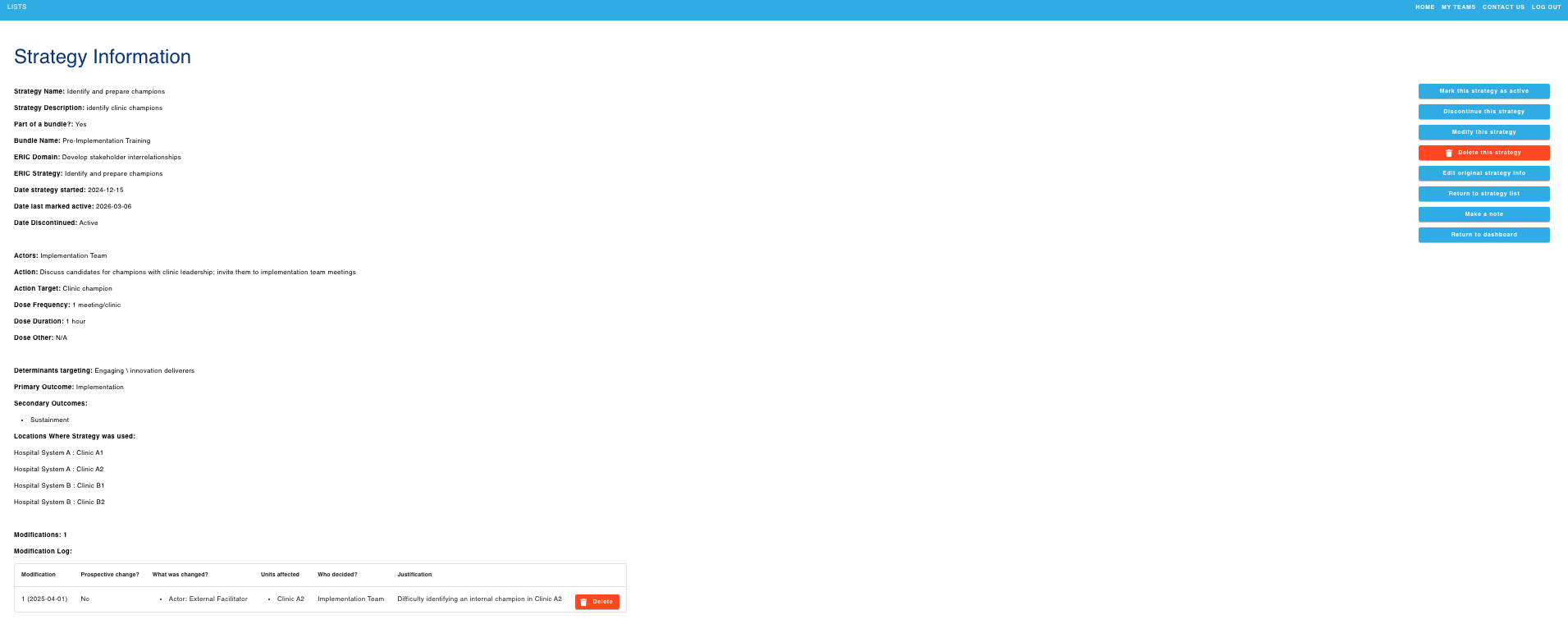


*Note*: Representative screenshot of the Strategy Information page, which displays detailed information for a selected strategy and supports ongoing tracking. Users can update strategy status, log modifications or discontinuations, and view a chronological record of changes, including associated notes and events..

Supplemental Figure 6. Strategy Modifications


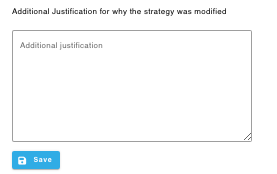

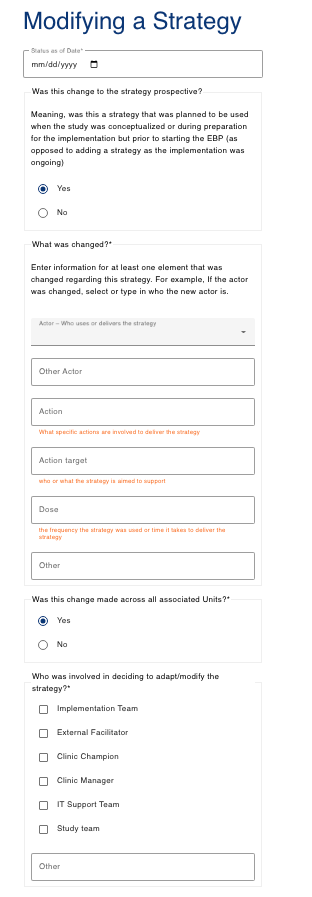

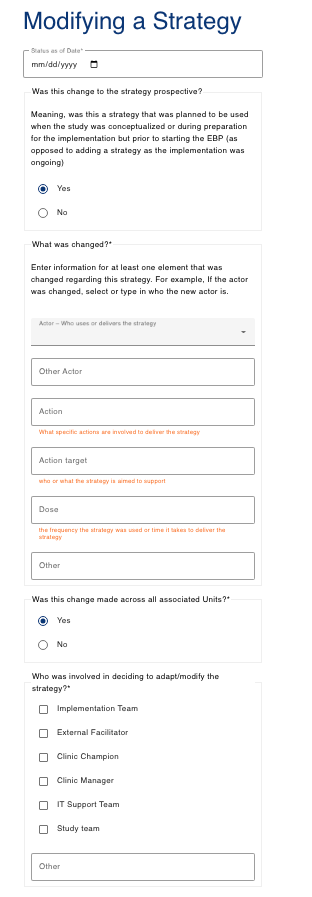


*Note:* Representative screenshot of the Strategy Modification interface, where users document changes to implementation strategies. Fields include modification date, whether the change was planned, elements modified, unit-level applicability, decision-maker(s), and justification. The discontinuation interface follows a similar structure–only the ‘What was changed’ section is omitted.
