# Supplementary material for: Usability and feasibility of the longitudinal implementation strategy tracking system: a think-aloud study with implementation researchers
Source: Front Health Serv. 2026 May 12;6:1837215. doi: 10.3389/frhs.2026.1837215 (PMC13201223; doi:10.3389/frhs.2026.1837215)
Supplement: Supplemnetary Table S1 — Codebook for Hybrid Deductive–inductive thematic analysis. [file Table1.docx]

Supplemental Table 1: Codebook for Hybrid Deductive-Inductive Thematic Analysis

| **Code Category** | **Code Name** | **Operational Definition** | **Inclusion Criteria** | **Exclusion Criteria** | **Representative Example** |
| --- | --- | --- | --- | --- | --- |
| **Usability Strengths** | Structured Longitudinal Tracking | Participant endorses LISTS’ ability to systematically document strategies and adaptations over time | Comments about filling methodological gap; value of modification/ discontinuation logs | General dashboard comments without longitudinal reference | “This really helps you see what changed over time.” |
|  | Conceptual Alignment with Frameworks | Participant values integration with ERIC, CFIR, RE-AIM, Proctor outcomes | Statements endorsing theoretical rigor | Terminology burden comments | “I like that this ties directly to ERIC categories.” |
|  | Dashboard Visualization & Export Utility | Participant endorses timeline, filtering, or export functionality | Statements about analytic usefulness; communication utility | Confusion about visual cues (coded separately) | “This would be really helpful for sharing with the team.” |
| **Conceptual & Terminology Friction** | Terminology Density | Cognitive friction due to implementation science terminology | Hesitation distinguishing constructs; comments about jargon | Navigation confusion unrelated to terminology | “I’m not sure how sustainment differs from sustainability.” |
|  | Fidelity–Accessibility Tension | Tension between theoretical rigor and broader usability | Calls for “LISTS Lite”; concerns about non-expert users | Isolated terminology confusion | “This assumes a baseline level of implementation knowledge.” |
| **Hierarchy & Structural Logic** | Multi-Level Unit Hierarchy Confusion | Difficulty specifying or interpreting nested organizational levels | Confusion during project setup; unclear hierarchy relationships | Non-hierarchical confusion | “I’m not sure how to distinguish hospital from clinic here.” |
|  | Unit-Specific Modification Limitation | Difficulty attaching modifications to specific units | Requests to link modifications to sites; workaround descriptions | General hierarchy confusion | “I’d want to specify that this change was only for Clinic B.” |
| **Modification & Audit Trail Workflows** | Editing vs Logging Modification Ambiguity | Uncertainty whether to edit original strategy or log modification | Concerns about overwriting; audit trail preservation | Simple interface navigation errors | “Am I supposed to edit this or log it as a change?” |
|  | Modification Log Visualization Complexity | Difficulty interpreting multiple sequential modifications | Comments about clutter or sequencing | Single modification confusion | “If there are five changes, this could get hard to follow.” |
|  | Justification Field Insufficiency | Recommendation to expand or enhance free-text justification field | Requests for more space or nuance capture | General modification confusion | “It would help to have more room to explain why this changed.” |
|  | Date Semantics Ambiguity | Confusion about whether dates reflect occurrence vs entry | Clarification questions about modification dates | General date entry errors | “Is this the date it happened or the date I’m entering it?” |
| **Interface Affordances & Visual Encoding** | Visual Encoding Ambiguity | Confusion about color coding, status indicators, or timeline representation | Uncertainty about active vs discontinued; timeline orientation issues | General usability complaints | “Why are these different colors? What does that mean?” |
|  | Required vs Optional Field Ambiguity | Unclear signaling of mandatory versus optional fields | Orange text interpreted as required; hesitation completing fields | Required-field errors unrelated to perception | “It looks like I need to fill all of these in.” |
|  | Button State & Action Clarity | Recommendation to disable, gray out, or relocate buttons | Delete button confusion; action placement suggestions | Simple misclicks | “This should probably be grayed out until it’s relevant.” |
| **Data Entry Integrity & Flexibility** | Actor Entry & Editing Concerns | Concerns about misspellings, duplicates, or inability to edit actors | Spellcheck requests; editing flexibility concerns | General typing errors | “Will I be stuck with this misspelled actor forever?” |
|  | Data Validation & Error Prevention | Suggestions to reduce entry errors and improve data quality | Input validation suggestions; duplication safeguards | General conceptual confusion | “It would be helpful if it flagged duplicates.” |
| **Workflow & Scalability** | Single-Screen Workflow Burden | Difficulty managing tool alongside reference materials | Comments about split-screen challenges; desire for import tools | General time burden comments | “It’s hard to do this with just one monitor.” |
|  | Target User Role Ambiguity | Uncertainty about who should manage LISTS | PI vs project manager discussions; workflow guidance | Terminology burden unrelated to role | “Who on the team would actually be responsible for this?” |
|  | Real-World Integration Challenges | Concerns about integrating LISTS into real implementation workflows | Comments about meeting integration; sustainability concerns | Usability comments unrelated to workflow | “You’d need to build this into standing meetings.” |
| **Protocol-Level Codes** | Think-Aloud Protocol Feasibility | Feedback on clarity, realism, or scalability of usability protocol | Comments about session length; vignette realism | Tool usability comments | “The vignette worked well, but this might be long for some users.” |
|  | Evaluation Instrument Feedback | Suggestions to refine SUS or post-session questionnaire wording | Rewording recommendations; evaluation clarity concerns | Tool usability comments | “Some of these survey questions feel unclear.” |
| **Cross-Cutting Meta-Code** | Interpretive Friction | Conceptual hesitation requiring interpretation without navigation failure | Pauses to interpret constructs; reflective hesitation | Task failure or error | “I need to think about what this construct really means.” |
