# Supplementary material for: Usability and feasibility of the longitudinal implementation strategy tracking system: a think-aloud study with implementation researchers
Source: Front Health Serv. 2026 May 12;6:1837215. doi: 10.3389/frhs.2026.1837215 (PMC13201223; doi:10.3389/frhs.2026.1837215)
Supplement: Supplementary Table S2 — Cross-case matrix for primary qualitative themes. [file Table2.docx]

Supplemental Table 2.

Cross-Case Matrix for Primary Qualitative Themes

**Key:** ✓ = Theme present in participant transcript
Blank = Not meaningfully observed

| **Primary Theme** | **P1** | **P2** | **P3** | **P4** | **P5** | **P6** | **P7** | **P8** | **P9** | **Total (n)** |
| --- | --- | --- | --- | --- | --- | --- | --- | --- | --- | --- |
| **Structured Longitudinal Tracking (Strength)** | ✓ | ✓ | ✓ | ✓ | ✓ | ✓ | ✓ | ✓ | ✓ | 9 |
| **Conceptual Alignment with Frameworks (Strength)** | ✓ |  | ✓ | ✓ | ✓ |  | ✓ |  | ✓ | 6 |
| **Dashboard Visualization & Export Utility (Strength)** |  |  | ✓ |  | ✓ |  | ✓ |  | ✓ | 4 |
| **Terminology Density (Limitation)** | ✓ | ✓ | ✓ | ✓ | ✓ | ✓ | ✓ | ✓ | ✓ | 9 |
| **Multi-Level Unit Hierarchy Confusion (Limitation)** | ✓ |  | ✓ | ✓ | ✓ | ✓ | ✓ | ✓ | ✓ | 8 |
| **Ambiguity Between Editing and Logging Modifications (Limitation)** |  |  | ✓ | ✓ | ✓ |  | ✓ | ✓ | ✓ | 6 |
| **Fidelity–Accessibility Tension (Cross-Cutting)** | ✓ |  | ✓ | ✓ | ✓ |  | ✓ |  | ✓ | 6 |

*Note:* Presence reflects meaningful contribution to theme during think-aloud or post-session reflection.
